# Current status on cyclotron facilities and related infrastructure supporting PET applications in Latin America and the Caribbean

**DOI:** 10.1186/s41181-022-00166-z

**Published:** 2022-06-13

**Authors:** Miguel A. Avila-Rodriguez, Amir R. Jalilian, Aruna Korde, David Schlyer, Mohammad Haji-Saeid, Jose Paez, Saul Perez-Pijuan

**Affiliations:** 1 Universidad Nacional Autónoma de México, Ciudad de México, 04510 México; 2 International Atomic Energy Agency, 1400 Vienna, Austria; 3 Brookhaven National Laboratory, Upton, NY 11973-5000 USA; 4 Instituto Peruano de Energía Nuclear, Lima, Perú

**Keywords:** Cyclotrons, Radionuclide production, PET radiopharmaceuticals, IAEA database of cyclotrons, Latin America & Caribbean

## Abstract

This review presents the results of a survey conducted by the International Atomic Energy Agency on cyclotrons and related infrastructure used for radionuclide and radiopharmaceutical production which are supporting PET imaging applications in Latin America and the Caribbean region.

## Background

Positron Emission Tomography (PET) is a proven useful tool for the diagnosis and management of oncological, neurological, and cardiological disorders, and it is widely used in current clinical practice (Kitson et al. 2009). The progress of PET has gone hand in hand with radiopharmacy, and thanks to the development of new radiopharmaceuticals directed to specific molecular targets that allow non-invasive visualization, characterization, and quantification of biological processes at the molecular and cellular levels, PET imaging has rapidly become the method of choice for the diagnosis and monitoring of many diseases in clinical settings (Anand et al. 2011). This imaging technique primarily relies on the availability of cyclotron-produced radionuclides and considering the short half-life (few minutes to several hours) of most of the positron emitting radionuclides (Nickles 2002), is imperative an on-site or nearby cyclotron facility within a convenient distance, reachable within 1–2 half-lives of the respective radionuclide (Saha et al. 1992). PET technology was developed in the late 1970s, however, it became more feasible in clinical settings only during the 1990s, thanks to the technology developments and commercialization of compact negative ions accelerating cyclotrons (Schmor 2011), which are now widely available. The International Atomic Energy Agency (IAEA) has recently updated the Database of Cyclotrons for Radionuclide Production (IAEA, 2022) which features approximately 1,300 cyclotron facilities for medical radionuclide production around the world. This IAEA’s database date back to 1983 and is updated and maintained by the Radioisotope Products and Radiation Technology Section, Division of Physical and Chemical Sciences.

The first compact cyclotron for PET radionuclide production in Latin America was installed in 1997 in Argentina at Fundación Escuela de Medicina Nuclear (Tutor and Frias 2002), followed by the installation of the second PET-cyclotron at Universidad Nacional Autónoma de México (UNAM) in 2000 (Avila 2001). At that time, the learning curve, assimilation, and implementation of new emerging technologies for PET applications was slow, but in the following years this innovative imaging technique captured the attention of the medical and scientific community from other countries in the region, and by 2010 over 15 more compact cyclotrons were already installed in Latin American countries. As per its mandate to promote peaceful uses of nuclear energy, the IAEA assists its Member States (MS) for capacity building/knowledge sharing, both in human resources and infrastructure development in related fields, including medical radioisotope production, radiopharmaceuticals and its applications through various activities. These activities are primarily training courses, workshops, technical meetings, co-ordinated research projects (CRPs), publications (technical documents, guidance documents, manuals, books etc.), training and teaching through web based educational content, conferences/symposia, expert missions, and individual hands-on training at established centers. The Technical Cooperation Programme is the primary mechanism for transferring nuclear technology to MS, helping them to address key development priorities in specific areas.

The State Parties of the Regional Cooperation Agreement for the Promotion of Nuclear Science and Technology in Latin America and the Caribbean (ARCAL), under the auspices of IAEA, undertake to promote, foster, coordinate, and implement cooperation activities for training, research, development and applications of nuclear science and technology. The IAEA’s Technical Cooperation Projects are usually of two- to four-year duration, focused to achieve specific objectives and outcomes; RLA6085 entitled “Strengthening the Capacities of Cyclotron/PET Centres in the Region” is one such recently approved ARCAL project. To serve as baseline for this ARCAL project, the IAEA conducted a survey on cyclotrons and related infrastructure used for production of radionuclides and PET radiopharmaceuticals, which are supporting PET imaging applications for patients in Latin America and the Caribbean region. The specific objective of this survey was to gather information on the number of institutions operating cyclotron facilities and related infrastructure, staff involved in the operation and maintenance of cyclotron as well as those in the production and quality control of radionuclides and radiopharmaceuticals. The survey considered the radionuclides and radiopharmaceuticals that are routinely produced and/or in various stages of development, including novel radiopharmaceutical in the near future (2–4 years) planning. The results of this survey are presented in this publication.

## Design and conduction of survey

A preliminary list of 57 cyclotron facilities in the region was obtained from the IAEA Cyclotron Database (IAEA, 2022), and the information was crosschecked with three main cyclotron manufacturers: GE Healthcare (GE), IBA RadioPharma Solutions (IBA), and Siemens Healthineers (Siemens). Given the limited number of cyclotrons from other manufacturers in the region (Advanced Cyclotron Systems Inc., ACSI; ABT Molecular Imaging Inc.; and The Cyclotron Corporation, TCC) it was not necessary to crosscheck the information with these companies. The manufacturers validated and updated the information on the cyclotrons in the region, including cyclotrons in operation, in progress of installation and in commissioning, as well as new cyclotron projects with a signed contract. This way, an updated list of cyclotron facilities in the region together with that of the institution, location (city and country), and cyclotron model was prepared. However, due to internal policies of confidentiality, the contact person information could not be obtained from the manufacturers. Sorting the cyclotron sites by country location was helpful for getting contact information for most of the cyclotron facilities in the region. Detailed information of cyclotron facilities was obtained using interregional professional contacts and networks.

To facilitate the response of the questionnaire, a Google Form was designed to perform an online survey with multiple option items to answer most of the entries. In all cases, individual and personalized email messages were sent to the contact persons of each cyclotron facility requesting their help/support to answer the survey. The survey was conducted in July 2021. Requested data included institution and contact person information, cyclotron characteristics, details of radiochemistry synthesis modules, radionuclide and radiopharmaceutical production and applications, equipment available for quality control and preclinical facilities. Other important aspects covered in the questionnaire were related to personnel including education and training, applicable Good Manufacturing Practices (GMP) and health regulations in the country.

### Cyclotron statistics and characteristics

**Fig. 1:**
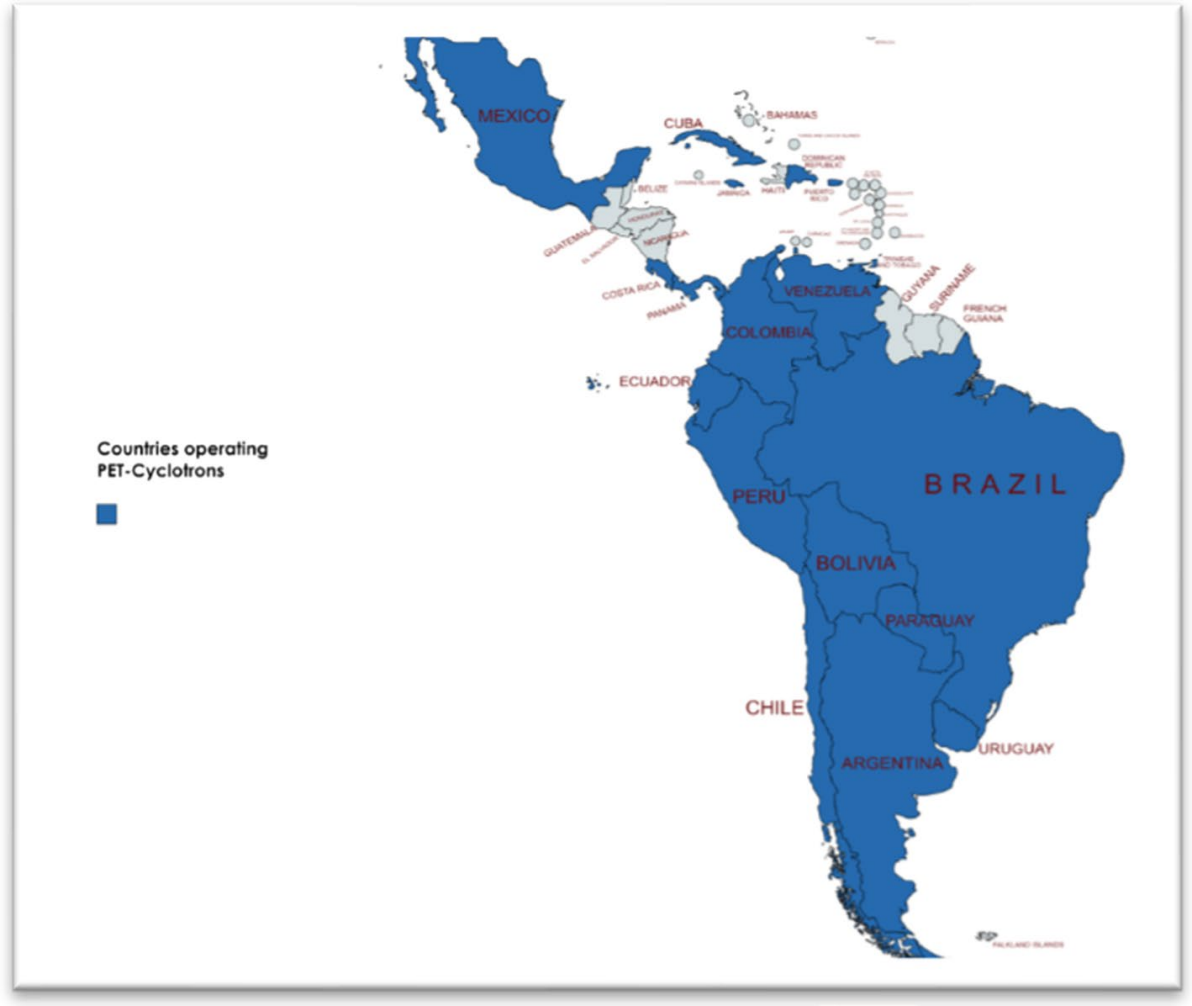
Latin America and Caribbean map of Member States operating cyclotrons for radionuclide production (Map created using mapchart: https://mapchart.net/)

The study confirmed the existence of sixty-seven cyclotrons in Latin America and the Caribbean region. The response to the survey was very positive, 46 out of 63 institutions completed the survey and the statistics presented in this report is based on the data provided by these responders. Figure 1 shows the map of Member States in Latin America and the Caribbean countries with cyclotrons for radionuclide production. The number of cyclotrons and estimated population in each MS is summarized in Table 1. Note that this table includes cyclotrons currently in operation (54), cyclotrons to be installed or under installation (3 in Argentina, 1 in Ecuador, 2 in Venezuela), in commissioning stage (2 in Argentina, 1 in Bolivia), inoperable (two BG-75 in Colombia, one Cyclone-18 in Mexico), and one RDS-111 cyclotron that is not in operating conditions and used only as a teaching tool in training courses for operators (Brazil).

**Table 1:** Number of cyclotrons by country and estimated population

Country	Estimated population 2021 (millions)*	Number of cyclotrons	Density of cyclotrons**
Argentina	45.2	10	0.22
Bolivia	11.7	2	0.17
Brazil	212.5	16	0.08
Chile	19.1	3	0.16
Colombia	50.9	6	0.12
Costa Rica	5.1	1	0.20
Cuba	11.3	1	0.09
Dominican Republic	10.8	2	0.19
Ecuador	17.6	3	0.17
Jamaica	3.0	1	0.33
Mexico	128.9	10	0.08
Panama	4.3	1	0.23
Paraguay	7.1	1	0.14
Peru	33.0	2	0.06
Puerto Rico***	2.9	2	0.69
Trinidad y Tobago	1.4	1	0.71
Uruguay	3.5	2	0.57
Venezuela	28.4	3	0.11

^*^https://www.worldometers.info/population, **Per million population, ***Officially an unincorporated territory of the USA

**Fig. 2:**
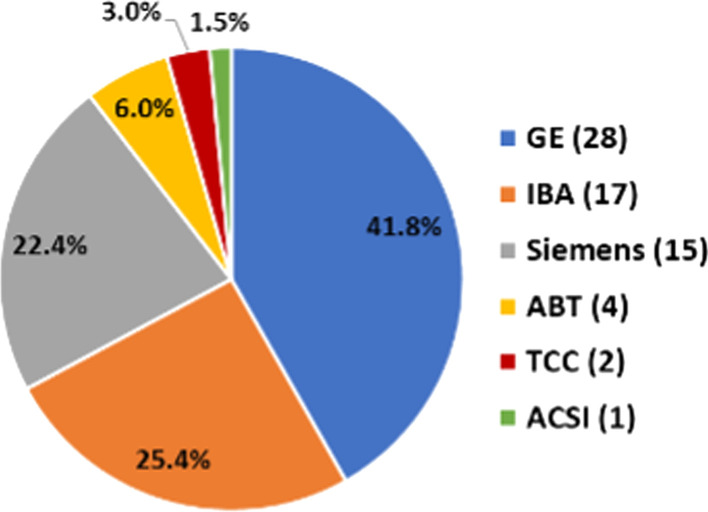
Distribution of cyclotrons sorted by manufacturer (n = 67)

Figure 2 shows the distribution of cyclotrons sorted by manufacturer and Table 2 provides details on their characteristics. Most of the cyclotrons in the region are obtained from any of the three main manufacturers (GE, IBA, and Siemens), maximum number (28 out of 67) of cyclotrons are from GE. Note: Siemens ceased the manufacturing of cyclotrons a few years ago.

**Table 2:** Number of cyclotrons by manufacturer, model, and proton energy

Manufacturer (total no. of cyclotrons)	Model	Proton energy (MeV)	Capability to generate other particle beams	No. cyclotrons
GE (28)	PETtrace	16.5	Deuterons	20
	MINItrace	9.6	N.A	7
	GENtrace	7.8	N.A	1
IBA (17)	Cyclone 18	18	Deuterons	10
	Cyclone KIUBE	18	Deuterons	4
	Cyclone 11	11	N.A	2
	Cyclone 30	30	Deuterons	1
Siemens (15)	Eclipse	11	N.A	11
	RDS 111			3
	RDS 112			1
ABT (4)	BG-75	7.5	N.A	4
ACSI (1)	TR24	24	Deuterons	1
TCC (2)	CV28	24	Deuterons, alpha	1
	CP42	42	N.A	1

N.A.: Not applicable

**Fig. 3:**
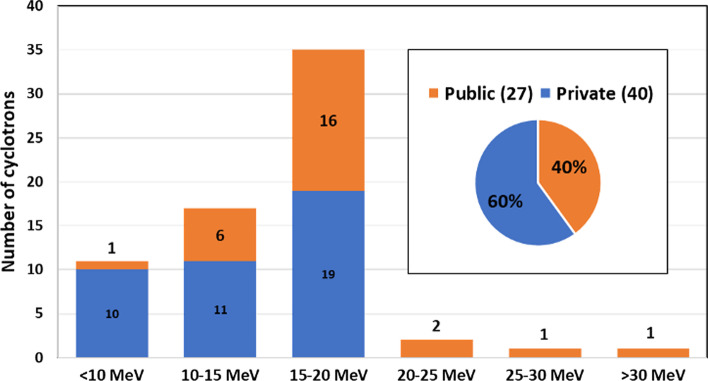
Distribution of proton energy for existing cyclotrons in the region (n = 67)

Among the existing cyclotrons in the region, 60% are owned by private institutions (non-State entity) and 40% belong to public hospitals/institutions (State owned entity). Figure 3 shows the distribution of cyclotrons based on proton energies The cyclotrons in the energy range of 10–15 MeV are predominantly manufactured by Siemens (11 MeV) while cyclotrons within energy range of 15–20 MeV are distributed between GE (16.5 MeV) and IBA (18 MeV) cyclotrons. Only four cyclotrons in the region are capable of generating proton beams with an energy > 20 MeV (TR24 in Bolivia, CV28 and Cyclone 30 in Brazil, and CP42 in Argentina), all of these are of variable energy types, whereas CV28 cyclotron is the only one with multiparticle acceleration capability (H, ^2^H and ^4^He). Seven institutions (2 private and 5 public) reported cyclotrons with dual-particle capability (H, ^2^H). Similarly, seven institutions reported availability of beam transport line(s), all of them installed in cyclotrons belonging to research centers or universities (public institutions).

**Fig. 4:**
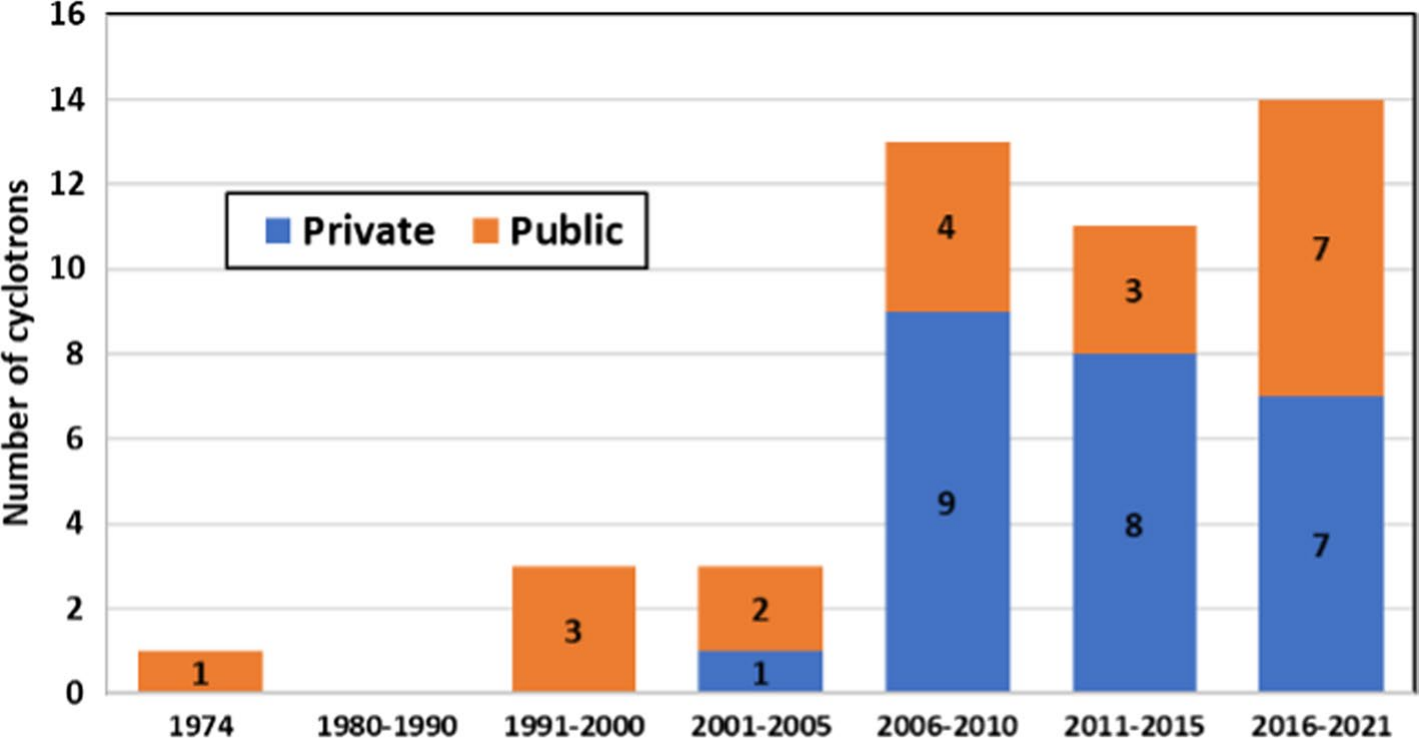
Distribution of cyclotrons by installation year (n = 45)

Figure 4 presents a distribution of cyclotrons by installation year. Note that this plot contains information of 45 out of the 67 cyclotrons confirmed in the region. Twelve institutions reported that their cyclotrons have been upgraded at least once (9 private, 3 public). Regarding cyclotron maintenance service 16 institutions (8 private and 8 public) reported the lack of annual maintenance service contract. Maintenance services are mainly provided by the companies that manufactured the cyclotrons.

### Radionuclide production

**Fig. 5:**
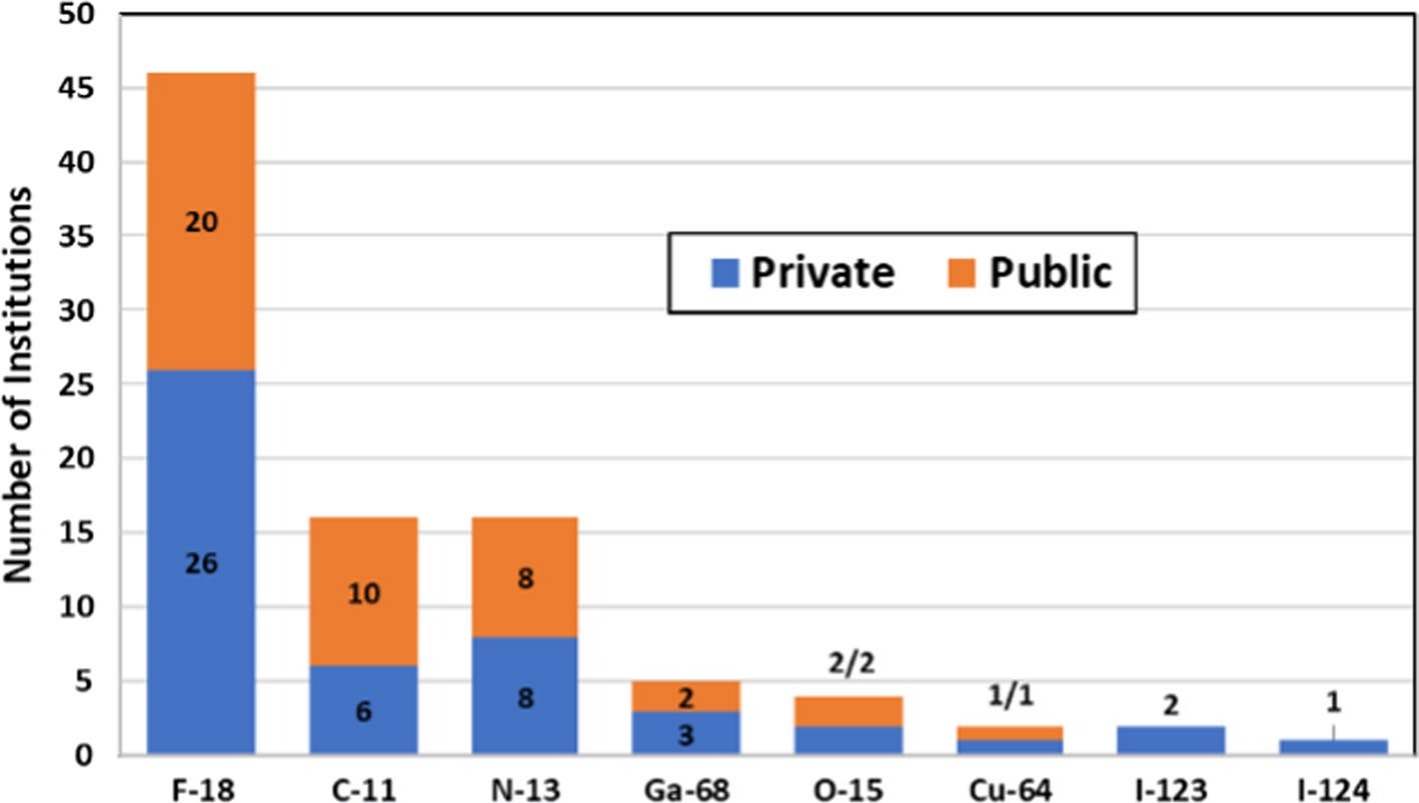
Number of institutions producing a particular radionuclide

All respondent institutions marked the production of fluoride-18 (F-18) followed by carbon-11 (C-11) and nitrogen-13 (N-13) as shown in Fig. 5. Only four facilities reported the production of oxygen-15 (O-15). Regarding the production of non-conventional PET radionuclides (radiometals and radiohalogens), five centers reported production of gallium-68 (Ga-68), four of them using solution targets and one using solid targets. Only two institutions in the region produce copper-64 (Cu-64), both in Mexico, while two institutions declared the production of radioiodine, one in Mexico (I-123 & 124) and one in Argentina (I-123).

### Radiopharmacy equipment and radiopharmaceutical production

**Fig. 6:**
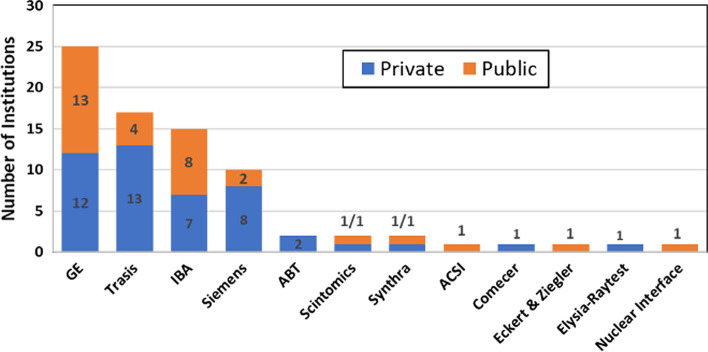
Distribution of chemistry modules by specific manufacturers

Radiotracer synthesizers (chemistry modules) in the region are predominantly from four manufacturers: GE, IBA, Siemens and Trasis (Fig. 6). Note that some facilities operate synthesizers from different manufacturers. Table 3 provides information on the models of chemistry modules and number of institutions in the region operating them.

**Table 3:** Distribution of chemistry modules by manufacturer and model

Manufacturer	Model	Number of institutions
GE	FASTlab*	17
	TRACERlab FX-C	8
	TRACERlab MX*	6
	TRACERlab FXFN	6
	TRACERlab FXFE	1
IBA	Synthera*	16
Siemens	Explora	11
Trasis	AllinOne*	16
	AllinOne mini*	2
	Easy One*	1
Comecer	Alceo	1
	Tadeo	1
Synthra	Unspecified	2
ABT	BG-75*	2
Scintomics	GRP*	1

*Chemistry modules operating based on single-use cassette basis

**Fig. 7:**
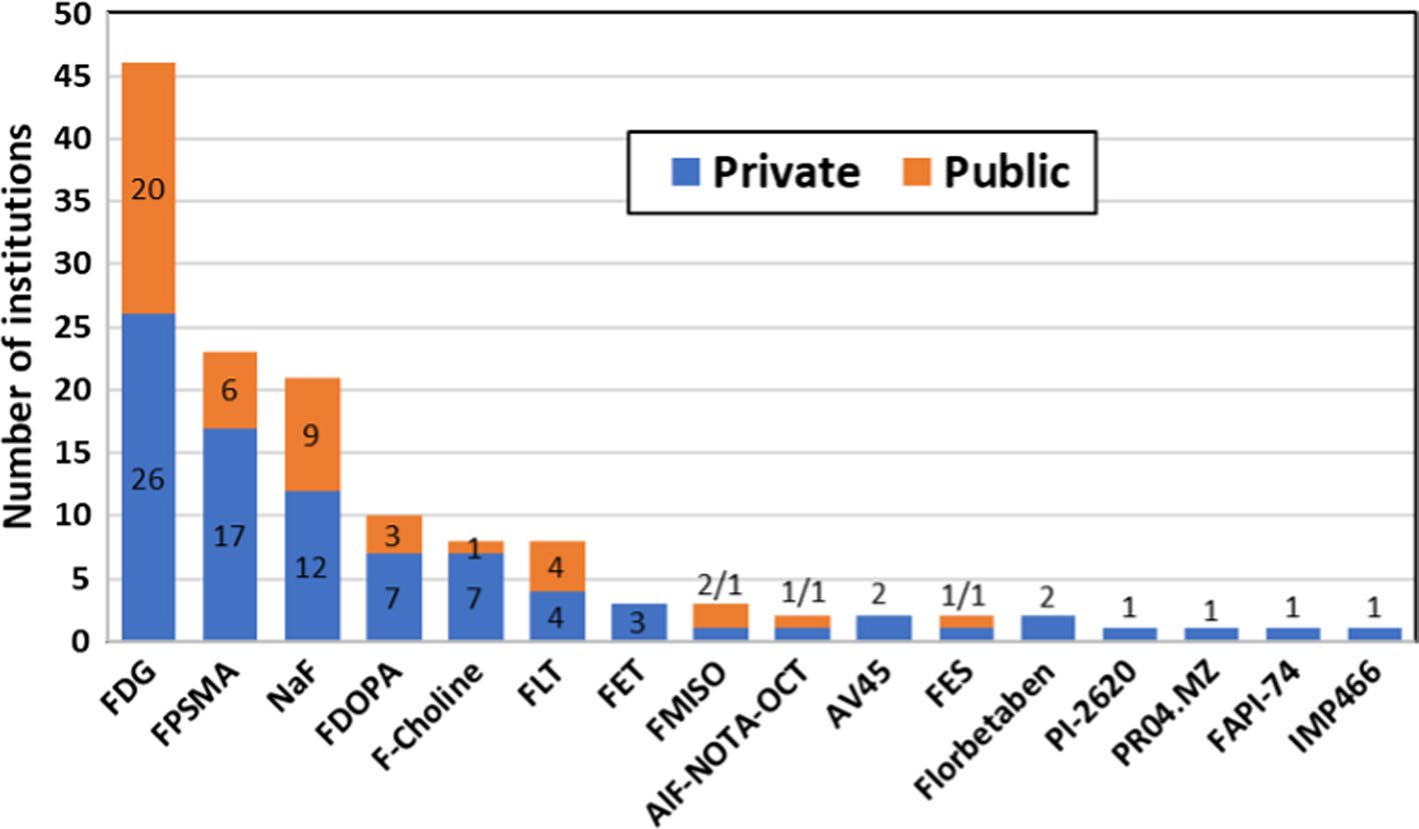
Main fluorine-18 radiopharmaceuticals produced in the region

**Fig. 8:**
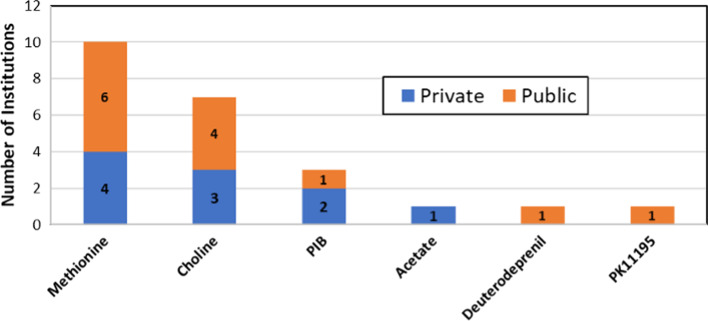
Main carbon-11 radiopharmaceuticals produced in the region

**Fig. 9:**
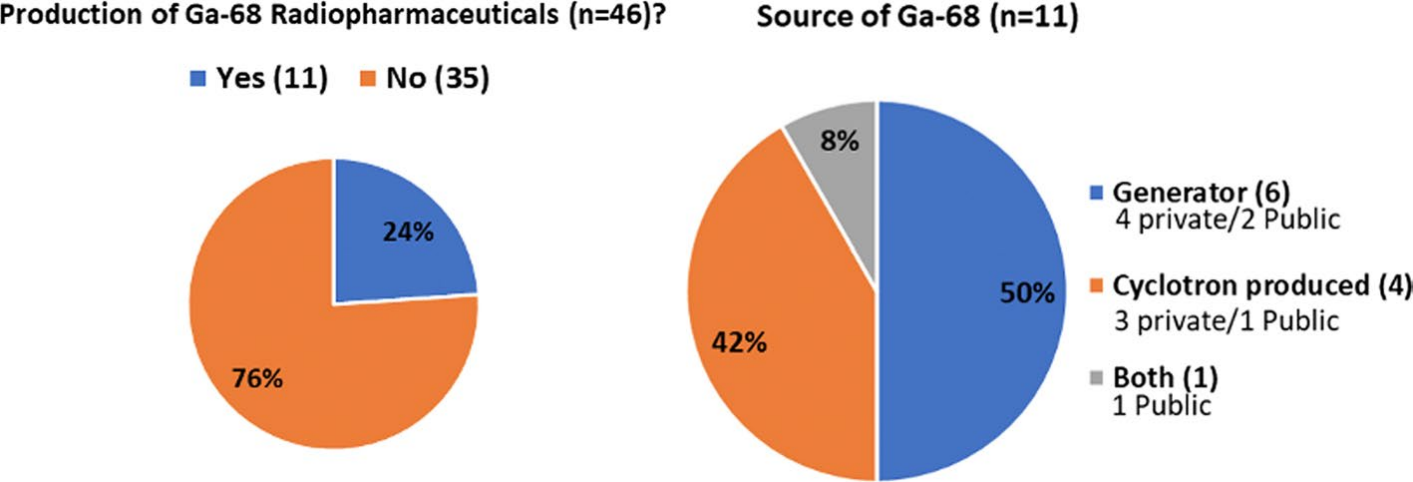
Institutions producing ^68^Ga-radiopharmaceuticals and the source of Ga-68

Figures 7 shows main F-18 labelled radiopharmaceuticals produced in the region. All facilities reported production of [^18^F]Fluorodeoxyglucose (FDG). After FDG, sodium fluoride (NaF) and ^18^F-labeled Prostate Specific Membrane Antigen (FPSMA) are the most commonly produced radiopharmaceuticals, while 6-[^18^F]Flurodopamine (FDOPA), [^18^F]Flurocholine (F-choline) and [^18^F]Fluorothymidine (FLT) are other commonly produced radiopharmaceuticals in the region. On the other hand, only 16 out of 46 respondent institutions disclosed the production of radiopharmaceuticals labelled with C-11. Methionine and choline are the most commonly produced ^11^C-labelled radiopharmaceuticals. Three facilities reported the production of C-11 Pittsburgh compound B (PIB) for amyloid imaging (Fig. 8). Only 24% (n = 11) of the facilities reported the production of ^68^ Ga-based radiopharmaceuticals and of those, six facilities reported the use of ^68^Ge/^68^ Ga generators, four facilities use cyclotron-produced Ga-68, and one facility uses both sources of Ga-68 as shown in Fig. 9. The majority of the modern tracer synthesizers are based on single-use cassettes and kits of reagents (see Table 3), which facilitates the production of a variety of radiopharmaceuticals, mainly labelled with F-18, under conditions that are compliant with GMP. This is not the case for C-11 radiopharmaceuticals, which is reflected in the limited number of facilities and tracers produced with this radionuclide; a similar situation still exists for Ga-68 radiopharmaceuticals. Most of the responses received to the specific question on the near future plans to produce new radiopharmaceuticals were related to radiolabeled enzyme inhibitors focusing mainly on PSMA and Fibroblast-Activation-Protein (FAP) compounds.

**Fig. 10:**
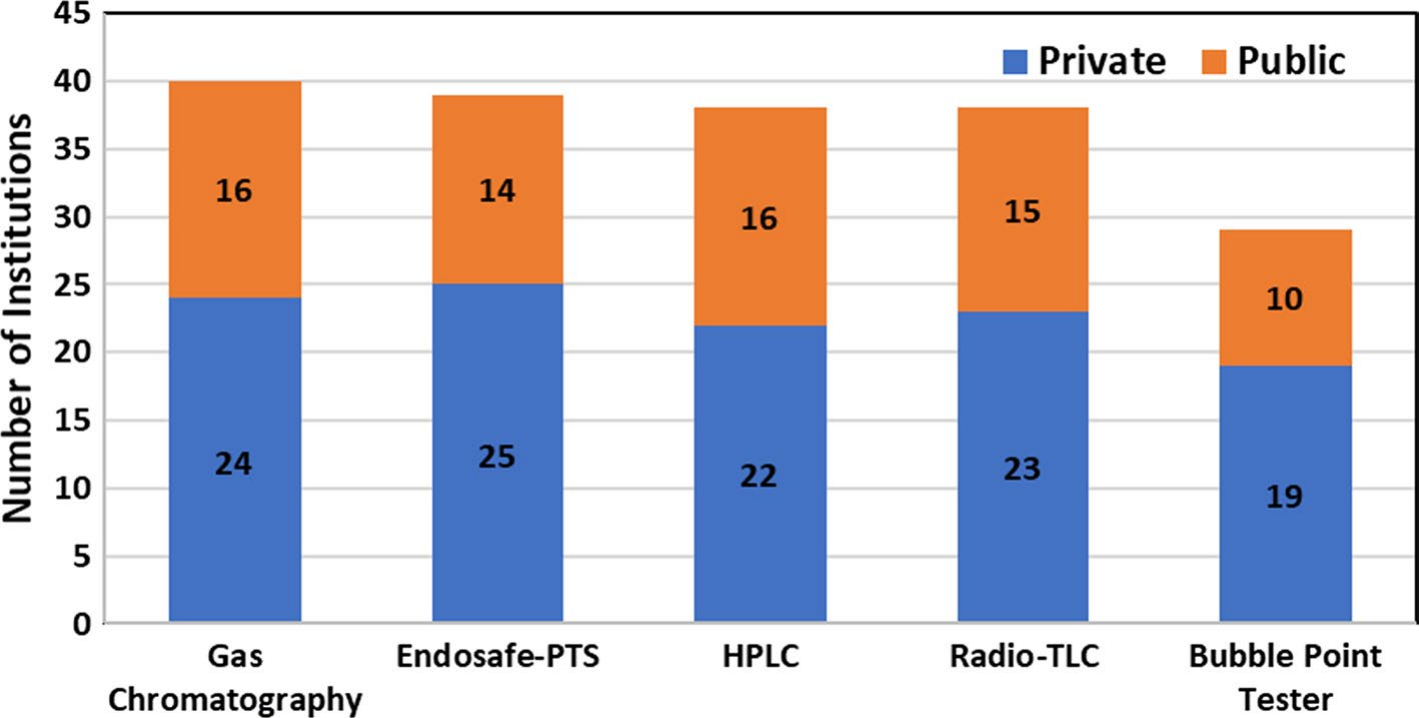
Institution’s reporting on availability of equipment for quality control of radiopharmaceuticals

**Fig. 11:**
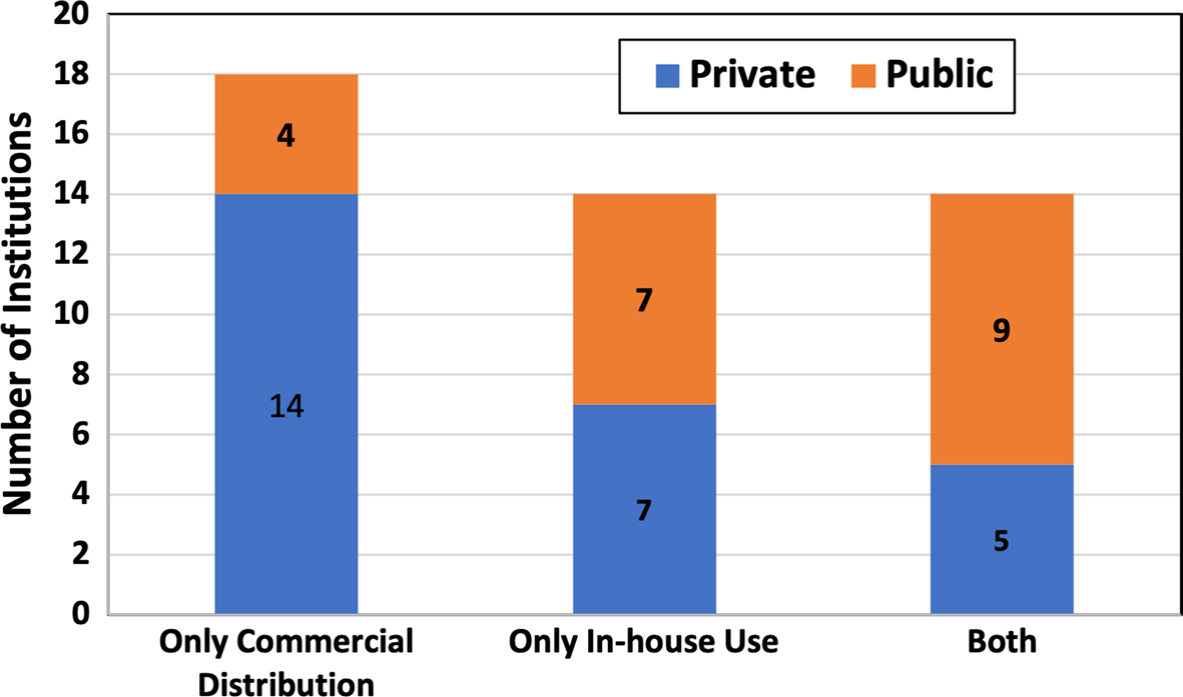
Radiopharmaceuticals distribution patterns in the region (n = 46)

The majority of production facilities proclaimed the availability of required essential equipment for quality control of radiopharmaceuticals as shown in Fig. 10. Eighteen institutions reported that radiopharmaceuticals are produced only for commercial distribution (40%), 14 produce radiopharmaceuticals only for in-house use (30%), and 14 more for both purposes (30%). Figure 11 depicts the percentage of production facilities with different distribution patterns for radiopharmaceuticals.

### Preclinical imaging

**Fig. 12:**
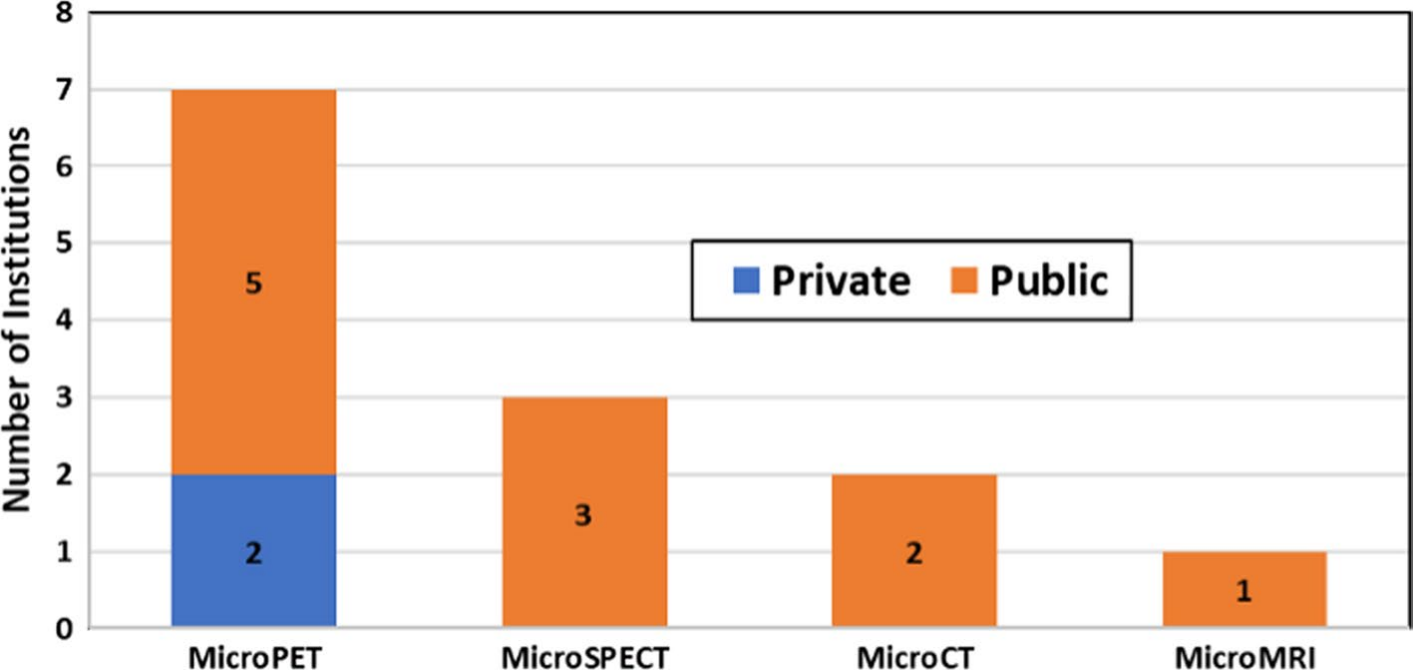
Institutions reporting preclinical imaging facilities with availability of different types of imaging modalities in the region

Seven institutions in the region divulged the existence of pre-clinical imaging facilities, with the availability of microPET equipment and other imaging modalities including microSPECT, microCT, and microMRI as shown in Fig. 12. Institutions with preclinical imaging equipment are mainly facilities belonging to universities and national centers or institutions with a strong component of research in their operations, all located in Argentina, Bolivia, Brazil, Mexico, and Uruguay.

### Human resources, education, training, and regulations

**Fig. 13:**
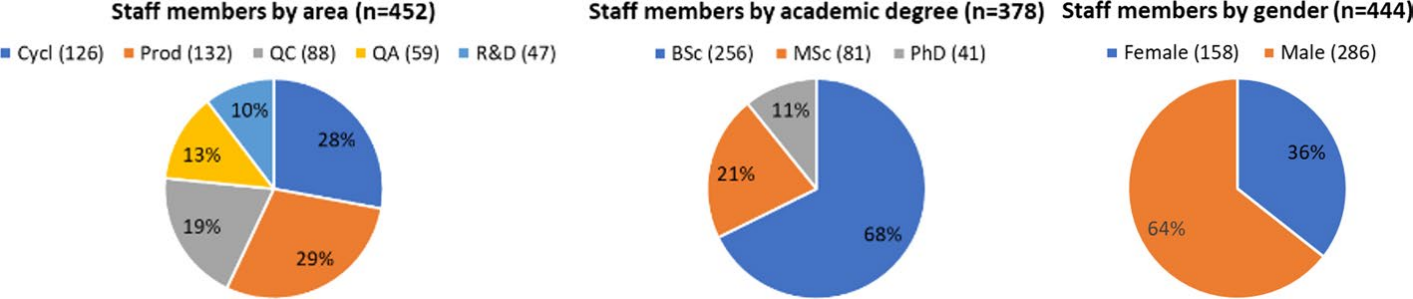
Important aspects of human resources of cyclotron facilities in the region

One important aspect of the survey was related to the human resources (HR) involved in the operation of cyclotrons and associated tasks in radiopharmaceutical production. The charts presented in Fig. 13 were generated using the total number of staff members (452) declared by respondents who completed the survey (n = 46). The first chart of staff members by area shows that most of personnel are involved in either cyclotron operation (126), production (132) and/or quality control (88) of radiopharmaceuticals. Fifty-nine staff members were declared to be involved in quality assurance tasks while forty-seven to be mainly involved in research and development (R&D) activities (16 in private and 31 in public institutions).

As for the distribution of academic degrees of personnel, the majority possess at least a bachelor's degree (256), followed by persons with a master's degree (81) and a doctorate degree (41). The survey did not ask for personnel without an academic degree, but from the difference of the total number of staff members working in the different areas (452), and the number of staff members with an academic degree (378), it could be inferred that 74 staff members do not have an academic degree. There were not significant differences between private and public institutions regarding personnel with bachelor and master’s degrees; however, of the 41 persons declared with a doctorate degree, 9 were from private institutions while 32 from public institutions. Regarding gender of staff members working in cyclotron facilities in Latin America and the Caribbean, there is a clear gender inequality as depicted in Fig. 13, with not significant differences between private and public institutions. The IAEA has undertaken some efforts to achieve gender parity; for instance, in 2019 the Network of Women in Radiopharmaceutical Sciences was launched as a professional network aiming at supporting, promoting, and empowering women in the field of radiopharmaceutical sciences. One year later the IAEA also launched the Marie Sklodowska-Curie Fellowship Programme that offers young women an opportunity to pursue studies towards a master’s in the nuclear field by providing financial support and practical experience. The aim of these initiatives is to help close the gender gap in the traditionally male-dominated nuclear sector. Additionally, the IAEA has always encouraged MS to preferably nominate suitable female candidates for participating in IAEA activities such as training programs, scientific visits, fellowships, various scientific and technical meetings.

**Fig. 14:**
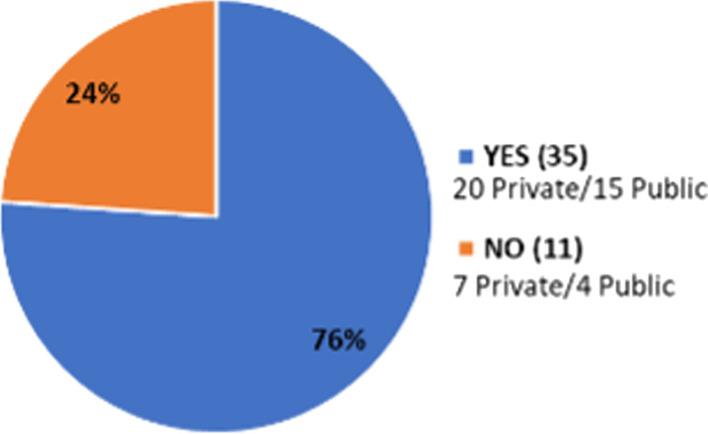
Availability of training/education programs in cyclotron facilities of the region (n = 46)

**Fig. 15:**
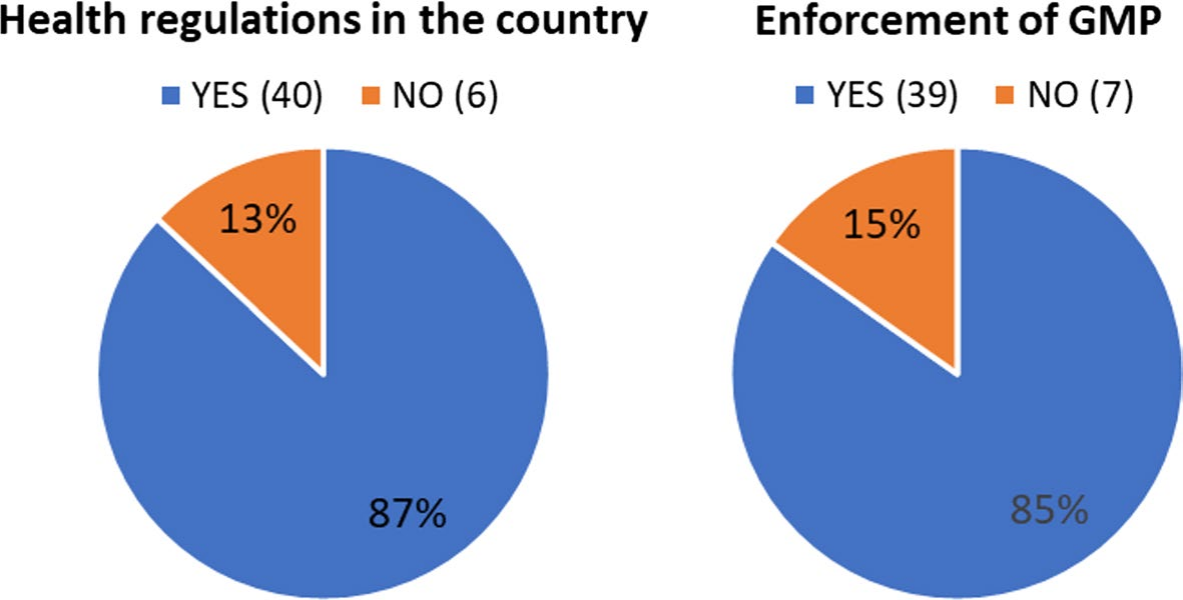
Facilities reporting health regulation in their countries (regarding radiopharmaceutical production and use) and enforcement of GMP (n = 46)

Thirty-five out of 46 cyclotron facilities that answered the survey declared the availability of a training/education program (Fig. 14). No further details were asked or given regarding the meaning of training/education programs but given the high number of institutions that declared their availability, the answers must be more probable related to GMP training requirements rather than formal programs on radiopharmaceutical sciences. On the other hand, Fig. 15 shows that most of the respondents noted down enforcement of GMP in their facilities (85%) and health regulation in their countries regarding radiopharmaceutical production and use (87%).

### Conclusions

Most Member States in Latin America operate at least one cyclotron. Of the seven countries located in the region of Central America, only Panama and Costa Rica count with a cyclotron, while of the Caribbean countries only five of them (Cuba, Dominican Republic, Jamaica, Puerto Rico, and Trinidad and Tobago) have cyclotrons. Qualified HR are essential to ensure the optimal operation of cyclotron facilities for radionuclide and radiopharmaceutical production for PET molecular imaging. The lack of trained and qualified HR is in some MS the limiting factor for the introduction of new technologies, and for the exploration of full potential that technology can offer and apply for the benefit of needy patients. One important aspect of the workforce of cyclotron facilities in the region is that most people working in the different areas (cyclotron operation, production, and quality control of radiopharmaceuticals) have at least a bachelor’s degree, facilitating the training in specific issues related to radiopharmaceutical sciences. IAEA projects, such as the recently approved RLA6085 and the ongoing RLA6084 ARCAL projects, can significantly contribute to strengthen human capacities and foster knowledge sharing in cyclotron operation, production, and quality control of PET radiopharmaceuticals.

## Data Availability

All data generated or analyzed during this study are included in this published article.

## Abbreviations

PET: Positron Emission Tomography
IAEA: International Atomic Energy Agency
MS: Member States
ARCAL: Regional Cooperation Agreement for the Promotion of Nuclear Science and Technology in Latin America and the Caribbean
GMP: Good Manufacturing Practices
HR: Human Resources
FDG: ^18^F-fluorodeoxyglucose
NaF: Sodium fluoride
FPSMA: ^18^F-labeled Prostate Specific Membrane Antigen
FDOPA: ^18^F-flurodopamine
F-choline: ^18^F-flurocholine
FLT: ^18^F-fluorothymidine
FET: ^18^F-fluorotyrosine
FMISO: ^18^F-fluoromisonidazole
FES: ^18^F-fluoroestradiol
FAPI: Fibroblast-Activation-Protein Inhibitor
